# Open-source pneumatic pressure pump for drop-based microfluidic flow controls

**DOI:** 10.1088/2631-8695/ace299

**Published:** 2023-07-12

**Authors:** Humberto S Sanchez, Connie B Chang

**Affiliations:** 1 Center for Biofilm Engineering, Montana State University, Bozeman, MT 59717, United States of America; 2 Department of Chemical and Biological Engineering, Montana State University, Bozeman, MT 59717, United States of America; 3 Department of Physiology and Biomedical Engineering, Mayo Clinic, Rochester, MN 55905, United States of America

**Keywords:** microfluidics, pressure-driven flow, open-source, drop-based microfluidics

## Abstract

An open-source pneumatic pressure pump is engineered for driving fluid flow in a microfluidic device. It is designed to be a cost-effective and customizable alternative to commercial systems. The pneumatic pressure pump utilizes a single open-source microcontroller to control four dual-valve pressure regulators. The control scheme is written in the Arduino development environment and the user interface is written in Python. The pump was used to pressurize water and a fluorinated oil that have similar viscosities. The pump can accurately control pressures to a resolution of less than 0.02 psig with rapid response times of less than one second, overshoot of desired pressures by less than 30%, and setting response times of less than two seconds. The pump was also validated in its ability to produce water-in-oil drops using a drop-making microfluidic device. The resultant drop size scaled as expected with the pressures applied to the emulsion phases. The pump is the first custom-made dual-valve regulator that is used to precisely control fluid flow in a microfluidic device. The presented design is an advancement towards making more fully open-source pneumatic pressure pumps for controlling flow in microfluidic devices.

## 1. Introduction

Microfluidics can translate biological and chemical assays from the lab bench to a device that is only several square centimeters in size [1]. These devices, commonly called microfluidic chips, are useful tools in many fields of biotechnology, particularly for single cell analyses [2]. Fluids are typically delivered into micron-sized channels on the chip from milliliter-sized vessels using mechanical syringe pumps [3], hydrostatic pressure pumps [4–6], or pneumatic pressure pumps [7–10]. Syringe pumps operate by converting the rotary actuation of a stepper motor into linear motion that drives the displacement of a syringe plunger [3]. Hydrostatic pressure pumps flow fluid from an elevated reservoir into the microfluidic channels, in which the pressure applied to the fluid is proportional to the height of the reservoir relative to the device [11]. Pneumatic pressure pumps utilize a pressure regulator to control the pressure of a compressed gas that drives fluid from a reservoir into the device [12, 13].

Pneumatic pressure pumps have several advantages over syringe and hydrostatic pumps. Syringe pumps have larger flow-rate fluctuations and slower response times to setpoint changes when compared to pressure pumps [11, 12]. Hydrostatic pumps have a narrower range of usable pressures and cannot be used for high-pressure applications as easily as pneumatic pumps [5]. Pneumatic pumps can also be used with a wider range of vessel volumes compared to syringe pumps.

Given the benefits of pneumatic pressure pumps for microfluidic research, engineering open-source versions of commercial pumps can be advantageous from a cost perspective, as commercial pumps can cost tens of thousands of dollars. An ideal open-source pump would consist of an open-source microcontroller and a customizable pressure regulator. The control scheme and its variables would be adjustable and uploaded to the microcontroller. The pump would be controlled using a guided user interface (GUI) written in an accessible programming language, such as Python [14]. Finally, the pump system would ideally not require custom machining or assembly.

Prior work creating open-source pneumatic pressure pumps utilized a blend of commercial and open-source components. Pressure pumps consist of four main components: the pressure regulator, the pressure transducer or sensor, the controller, and the GUI. Frank *et al* used commercially available parts for all of these components, except for the interface [8]. Gao *et al* developed a pump that used a commercial pressure regulator; the other components were open-source with a GUI written in C++ [10]. Finally, Watson and Senyo created a fully open-source pump with custom pressure regulators and a GUI written in C++. However, the pump created by Watson and Senyo was used to control valves on a microfluidic chip, not to drive flow [9].

In this work, we present a fully open-source pneumatic pressure pump designed to drive flow on a microfluidic device. Our pump utilizes a single open-source controller to control four dual-valve pressure regulators. The control scheme was written in the Arduino development environment and the GUI was written in Python. The ability of the pump to accurately change and regulate pressure was evaluated with step changes in pressure both without flow and when driving flow in a drop-making microfluidic device. The fluids used in these experiments were water and a fluorinated oil which have similar viscosities. When driving flow, the pump overshot the target pressures by less than 30%, settled within 5% of the desired pressure in less than two seconds, and was accurate down to less than 1% of the target pressure. The performance of the pump was comparable to previously reported open-source pneumatic pumps and other pumps. The pump performance was also validated in its ability to produce water-in-oil drops using a drop-making microfluidic device. The resultant drop size, measured by normalized drop length, scaled as expected to the applied pressures. This work demonstrates the utility of this custom built open-source pneumatic pressure pump for rapidly creating drops with stable fluid flow.

## 2. Method

### 2.1. Pneumatic design of the pressure pump

**Figure 1.**
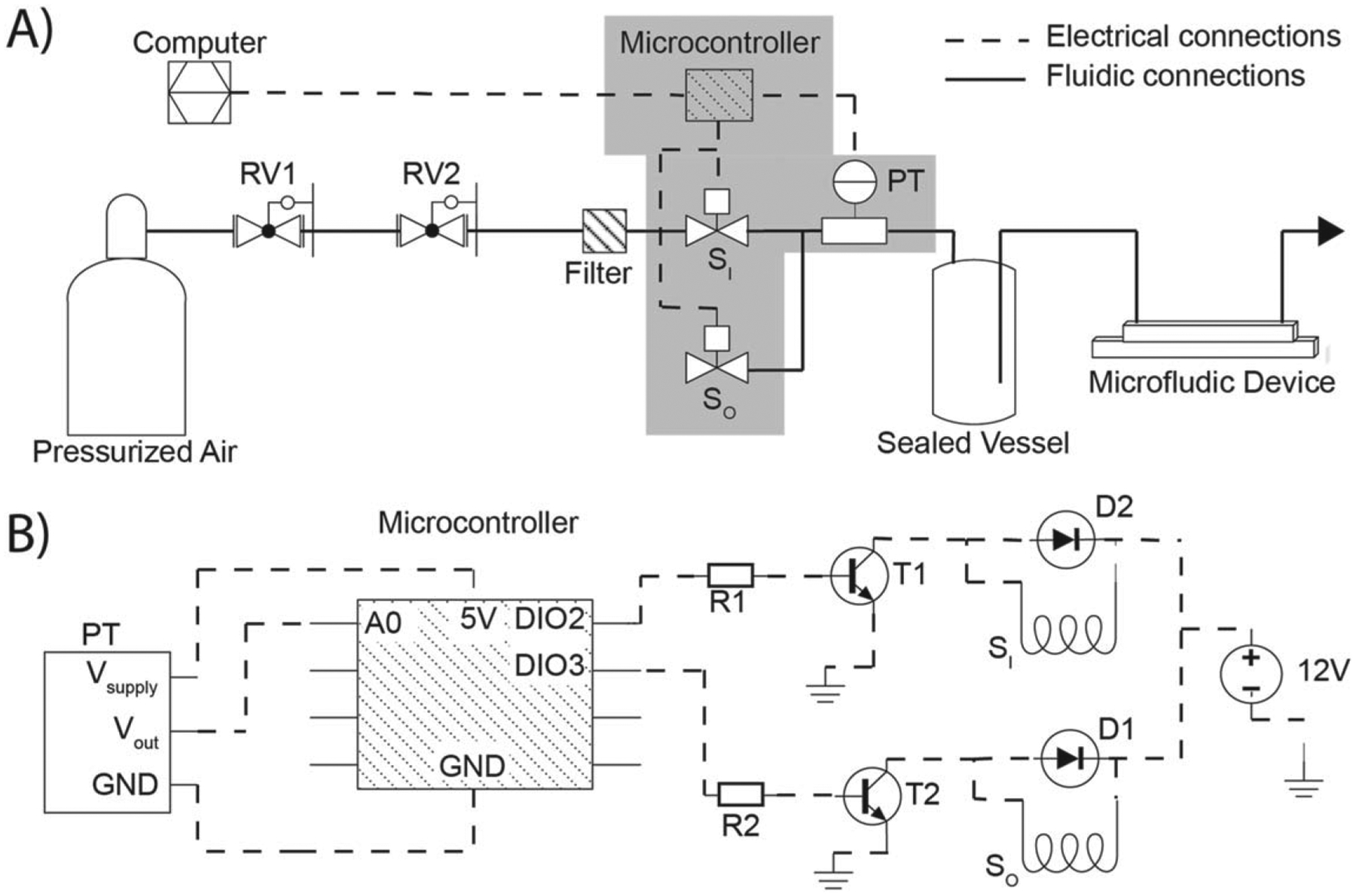
Diagram of mechanical and electrical components of the pneumatic pressure pump. (A) Mechanical component diagram of a dual-valve regulator. A compressed air tank supplies a high-pressure source which is adjusted using two regulator valves, RV1 and RV2. Two proportional solenoid valves, S_I_ and S_O_, regulate the pressure of a sealed vessel. One valve regulates air flow between a compressed air tank and the vessel, S_I_, and another regulates air flow from the vessel to atmospheric pressure, S_O_. The microcontroller controls each solenoid valve while monitoring the sealed vessels with a pressure transducer, PT. Liquid from the sealed vessel can then flow through a microfluidic device. Dashed and solid lines represent electrical and fluidic (gas or liquid) connections, respectively. (B) Circuit diagram, expansion of the grey region in (A). The PT sends an analog value proportional to the pressure to the pin A0. Pins DIO2 and DIO3 send PWM signals to the transistors T1 and T2 via base resistors R1 and R2. The PWM signals turn the solenoid valves, S_I_ and S_O_, on and off. The diodes, D1 and D2, protect the transistors during operation. Dashed lines represent electrical connections.

**Figure 2.**
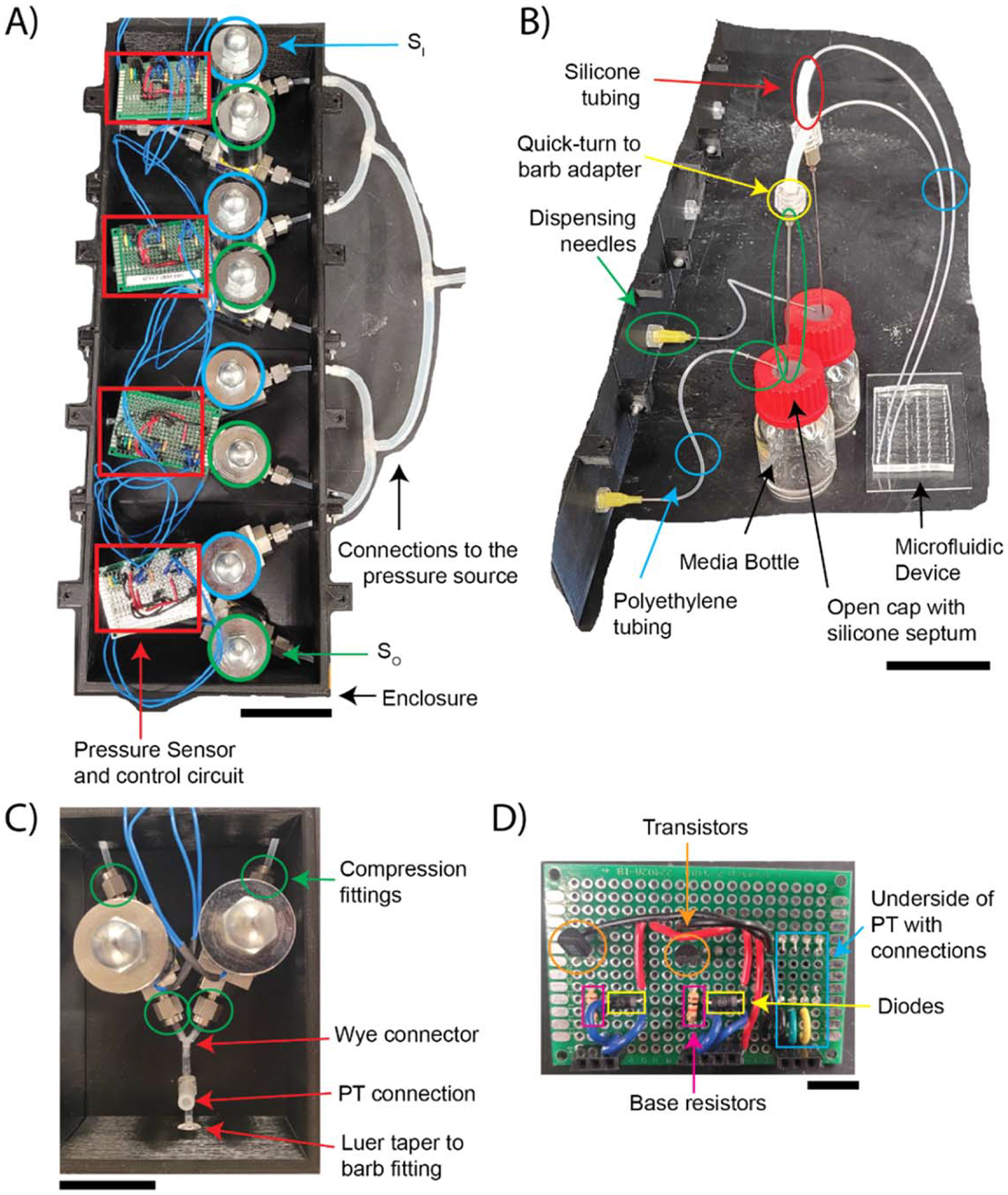
Images of different components of the pneumatic pressure pump. (A) A top-down view of the pneumatic pressure pump in the enclosure with the upper portion removed. The pressure sensors and control circuits (red), inlet solenoids (blue), outlet solenoids (green), the enclosure, and the tubing between the pump and the pressure source are highlighted. Scale bar = 5 cm. (B) A representative image of the connections between the pump, sealed vessels, and the microfluidic device. The various dispensing needles used to connect components are circled in green. The polyethylene tubing that connects the pump to the sealed vessel and the sealed vessel to the device are circled in blue. The quick-turn barb adapter and short length of silicone tubing that connect the sealed vessel to the microfluidic device are circled in yellow and red respectively. The media bottle, open cap with a silicone septum, and a microfluidic device are also indicated. Scale bar = 5 cm. (C) A representative image of the pneumatic connections inside the enclosure. The compression fittings of the inlets and outlets of the solenoid valves are circled in green. The wye connector that couples the solenoids, the connection for the pressure transducer, PT, and the luer taper to barb fitting that exits the enclosure are indicated with red arrows. The scale bar is 3 cm. (D) A representative image of the control circuit soldered onto the perfboard. The Transistors (orange), base resistors (magenta), the underside of the PT (blue), and the diodes (yellow) are outlined. Scale bar = 1 cm.

This pneumatic pressure pump utilizes a dual proportional solenoid design (figures 1 and 2). A compressed air tank is used as a positive pressure source for the pump design. A tank mounted regulator valve RV1 (McMaster-Carr, 7897A59) is used to set the pressure to ≈14 psig. A push-to-connect fitting (McMaster-Carr, 51235K107) is coupled to the downstream side of RV1 with a brass fitting (McMaster-Carr, 4429K111). Nylon tubing (McMaster-Carr, 5548K84) connects RV1 to an additional regulator valve, RV2, (McMaster-Carr, 9892K11) via an additional push-to-connect fitting. RV2 gives a more precise measurement of the pressure upstream of the pump. RV2 uses a barbed connector (McMaster-Carr, 5463K438) to couple 0.125-inch inner diameter silicone tubing (McMaster-Carr, 51845K53) to the remaining downstream connections. The silicone tubing is connected to a 25 mm 0.2 *μ*m filter (Fisher Scientific, GVS ABLUO^™^) with a luer taper to barb fitting (McMaster-Carr, 51525K273) to prevent particulates from entering the pump. The filter outlet connects to all the inlet solenoids S_I_ with more 0.125-inch silicone tubing and three wye connectors (McMaster-Carr, 53415K143). After these wye connectors, the tubing enters the enclosure that houses the solenoid valves. The solenoids use compression fittings upstream and downstream of the valve, figure 2(C). Nylon tubing (McMaster-Carr, 5548K81) is coupled to the 0.125-inch silicone tubing with a reducing adapter (McMaster-Carr, 5463K48). The S_I_ is coupled to the S_O_ with a wye connector, figure 2(C), (McMaster-Carr, 53415K143). The S_O_ uses the nylon and silicone tubing to vent outside of the enclosure. The pressure transducer (PT) (Honeywell, HSCDANN005PGAA5) couples downstream of the wye connector with a tee connector (McMaster-Carr, 5116K183), figure 2(C). The PT is connected to the upward facing barb and the downstream side of the tee is connected to a luer taper to barb fitting (McMaster-Carr, 51525K273) that exits the enclosure, figure 2(C).

### 2.2. Pneumatic interface between the pressure pump and a microfluidic device

The pump can be used to pressurize fluid in a sealed vessel (figure 1(A)). As a proof of concept, a 25 ml media bottle (Pyrex^™^, 139525) with an open cap (Corning, 1395–25HTSC) and a silicone septum (Corning, 1395–25SS) is used as the vessel, figure 2(B). The septum is pre-punctured with a 20-gauge needle (BD, 305176) in two locations for the pressurized inlet and the liquid outlet. The pressurized inlet is coupled to the pump with polyethylene tubing (Scientific Commodities, BB31695-PE/5) where one end is connected to a 20-gauge dispensing needle (McMaster-Carr, 75165A677), which is attached to the luer taper of the pump, figure 2(B). The other end of the polyethylene tubing is connected to an angled 20-gauge dispensing needle (McMaster-Carr, 75165A688) with the yellow hub removed and the shaft inserted into the polyethylene tubing. The liquid outlet uses a 6-inch-long stainless-steel dispensing needle (McMaster-Carr, 6710A85) with a quick-turn to barb adapter (McMaster-Carr, 51525K141), figure 2(B). The barb adapter is coupled to a short length of 0.79 mm inner diameter medical grade silicone tubing (Scientific Commodities Inc., BB519–13) followed by more polyethylene (Scientific Commodities Inc., BB31695-PE/2) tubing, figure 2(B). The polyethylene tubing is then coupled to another angled 20-gauge dispensing needle with the hub component removed. The end of the angled dispensing needle can then be inserted into the punched inlet of a PDMS microfluidic device.

### 2.3. Electrical design of the pressure pump

All four dual-valve pressure regulators are controlled from a single microcontroller using transistor switches. The circuit diagram used for all the pressure regulators is shown in figure 1(B). The PT transmits the measured pressure downstream of the solenoids to the microcontroller (Arduino, Mega 2560 Rev 3) via an analog signal. The microcontroller supplies the PT with 5 V and the PT transmits a voltage signal between 0 and 5 V which is proportional to a pressure between 0 and 15 psig. Alternative pressure sensors with varying ranges can be used in this setup depending upon the application. The microcontroller opens and closes the solenoids valves (Aalborg, PSV1S-BB) using pulse width modulation (PWM) enabled digital pins. The PWM pins send a power cycle (PC) that opens or closes the valves. The PC is sent to NPN transistors (ON Semiconductor, 2N4124) via base resistors (Digikey, 13-CFR-25JR-52–1KTR-ND, 1 kΩ ± 5%), figure 2(D). The transistors act as digital switches to power the solenoids with a 12V power supply (Mean Well, LRS-100–12). Diodes (ON Semiconductor, 1N4005G) are wired in parallel to the solenoids to provide the current stored in the inductive load a path to ground without shorting the transistor when the circuit is opened [15], figure 2(C). The electrical circuit was assembled and soldered onto a perfboard, figure 2(D).

### 2.4. Control scheme of the pressure pump

The feedback loop for the pressure regulators uses proportional control over the solenoid valves power cycles. If the measured point is lower than the set point, the inlet solenoid PC is increased by 0.004% and the outlet solenoid PC is decreased by the same amount. Conversely, if the measured point is higher than the set point, the inlet solenoid PC is decreased by 0.004% and the outlet solenoid PC is increased by the same amount. The increment and decrement values are the lowest gain available for the microcontroller based on the PWM resolution limit. This feedback loop operates continuously with a delay time of 1 ms between each iteration. The control scheme and serial communication software for the Arduino microcontroller is available on GitHub (https://github.com/thechanglab).

### 2.5. Guided user interface of the pressure pump

**Figure 3.**
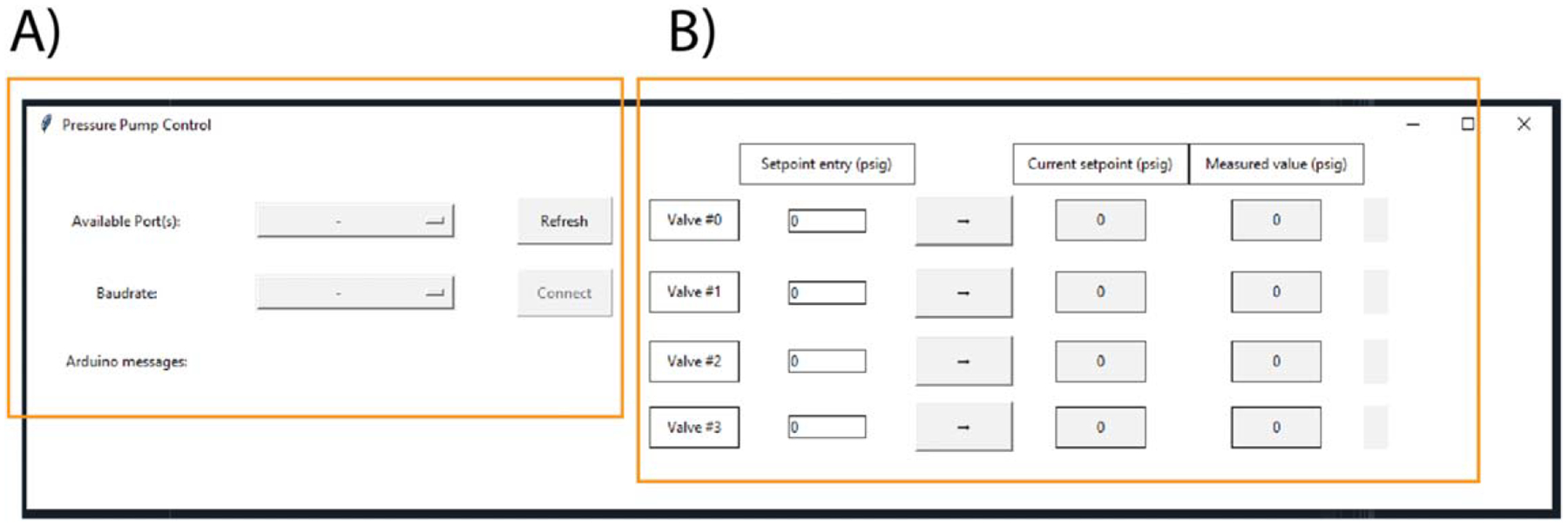
GUI used to control the pneumatic pressure pump. (A) This section detects peripherals connected to computer, selects the baud rate for serial communication, and connects to the microcontroller. (B) This section allows the user to change the set points of the four regulators, displays the measured points, and confirms that the change has been accepted by the microcontroller. The window size is 1250 by 300 pixels.

The pump interface is written in Python and allows users to control the system via a local computer and is shown in figure 2. The GUI was written in Python using the Tkinter and PySerial libraries [14]. The GUI is broken down into two main sections, one for establishing serial communication (figure 3(A)) and one for controlling the pressure regulators (figure 3(B)). Serial communication is established after selecting the serial port to which the pump is connected and selecting the baud rate that is used in the microcontroller. The *Refresh* button is used to detect all used serial ports. The *Connect*/*Disconnect* button establishes serial communication and the ‘*Arduino is ready to start*’ message is displayed if the correct parameters are selected. The set points of the individual regulators are changed after entering a new value in the ‘*Set point entry*’ column and then pressing the arrow button to the right of that column. The new set point is the displayed in the ‘*Current set point*’ column and the indicator to the far right of the GUI changes to green if that change has been accepted by the microcontroller. The current measured point is also displayed for each regulator. The GUI software is also available on GitHub (https://github.com/thechanglab).

### 2.6. Pressure regulation variables

The performance of the pressure pump was evaluated by applying changes in the set point and recording the measured pressures, or measured points. The pump performance was initially tested in the sealed vessel without liquid flow. The ability of the pump to regulate pressure is based off of four values: the overshoot (*OS*), the rise time (*t*_R_), the settling time (*t*_S_), and the accuracy [16]. The *OS* is determined graphically and is the percentage of how much the measured point goes over or under the new set point. Values of *t*_R_ and *t*_S_ are also determined graphically and are values of how much time is required for the measured point to reach the new set point and how long it takes for the measured point to be within 5% of the new set point. Accuracy is the average difference between the measured point and set point for all data points between the *t*_R_ and the time of another set point change.

### 2.7. Validation with microfluidic drop production

The pump performance was validated during microfluidic drop production. Drops are formed in a microfluidic device that uses a flow-focusing geometry to produce an emulsion comprised of an immiscible dispersed phase in a continuous phase stabilized by a surfactant [17]. The dispersed phase consisted of deionized water and the continuous phase consisted of 3% (w/w) ammonium carboxylate perfluoropolyether surfactant [18] dissolved in fluorinated oil HFE-7500 (3M). The drop-maker had a channel height of 40 *μ*m with an exit channel width of 100 *μ*m and was fabricated using soft lithography [19]. The channels were rendered fluorophilic with an injection of a solution of (tridecafluoro-1,1,2,2-tetrahydrooctyl) trichlorosilane (1 v/v%) in HFE-7500 (3M).

Pump performance can be assessed from its ability to form drops of different lengths proportional to the applied pressures. The major axis length of a drop in a microfluidic device (*ℓ*_*d*_) can be normalized to the width of the microfluidic channel (*w*_*ch*_) to give a dimensionless drop size. Ward *et al* has shown that this drop size is proportional to the squared ratio of dispersed and continuous phases pressures, *P*_D_ and *P*_C_ respectively, multiplied by a scalar value [20]. This relationship is shown in equation (1), where *α* is the scalar multiplier that can be determined using linear regression.


(1) $$\frac{\ell_{d}}{w_{ch}}=\alpha {\left(\frac{P_{D}}{P_{C}}\right)}^{2}$$

Drop sizes were determined during drop production using brightfield microscopy. During the drop production process, the microfluidic device was mounted on an inverted microscope (Nikon, TE2000, 10X objective lens). Footage of the drop-making process was captured using a high-speed camera (Edgertronic, SC2) at 5,000 frames per second. The footage was analyzed using drop morphometry and velocimetry (DMV) software [21]. The DMV software is specifically designed to analyze drops in microfluidic devices and allows the user to determine the time-history of drop lengths, widths, areas, positions, velocities, and pixel intensities.

Pump performance was also evaluated with step changes during microfluidic drop production. The *OS*, *t*_R_, *t*_S_, and accuracy were determined during step changes to the pressures applied to the continuous phase, *P*_*C*_.

## 3. Results

### 3.1. Pressure regulation without flow

**Figure 4.**
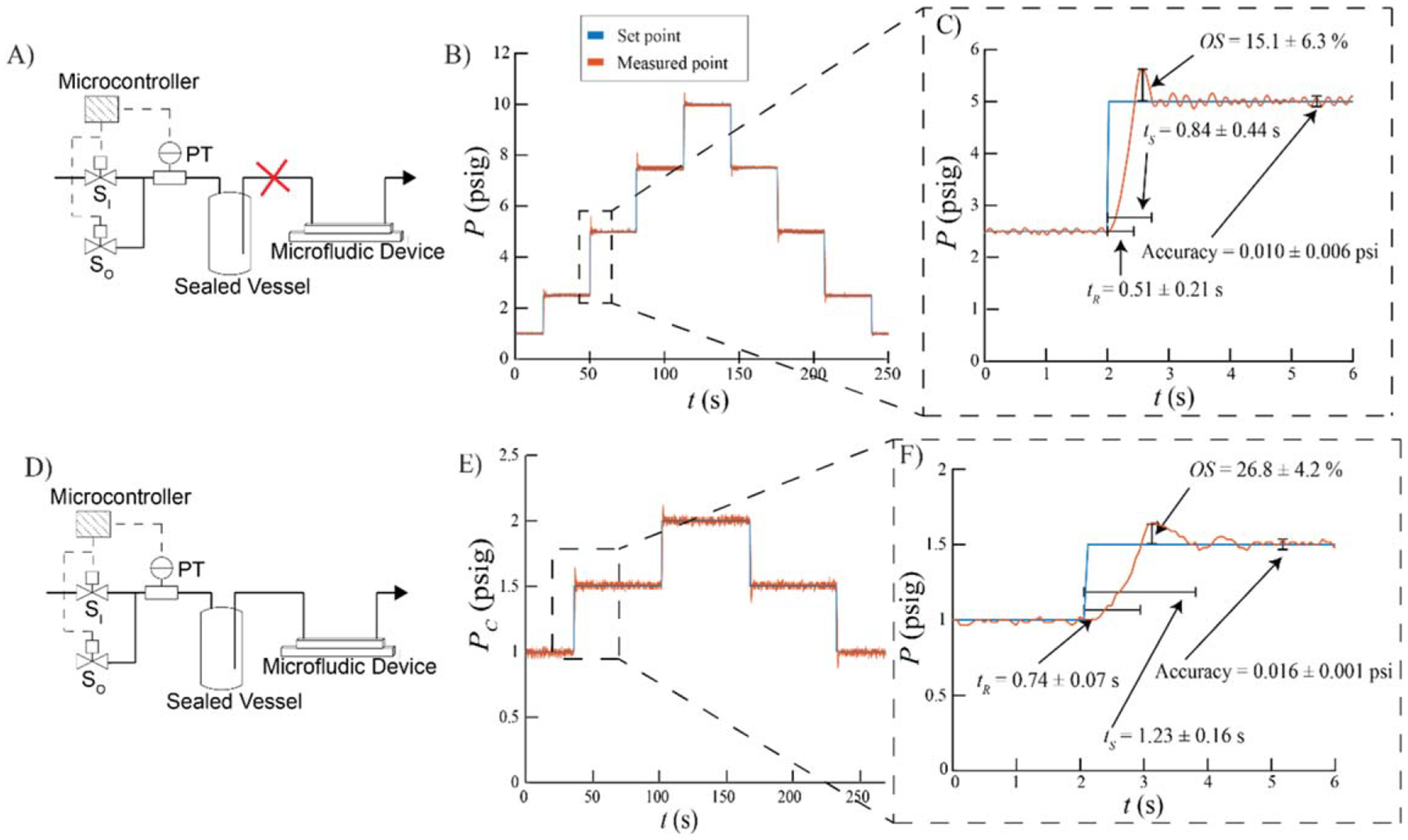
Response of the pneumatic pressure pump to step changes in set point without (A)–(C) and with outlet flow (D)–(F). (A) The pump schematic without outlet flow. (B) The set points (blue) and measured points (orange) of one pressure regulator in response to step changes. (C) A detailed zoom of the boxed region in (B) with the average rise time *t*_R_, settling time *t*_S_, overshoot *OS*, and accuracy shown. (D) The pump schematic connected to a microfluidic device. (E) The set points (blue) and measured points (orange) of the pressure regulator in response to step changes in the continuous phase pressure, *P*_C_, during microfluidic drop-making. The dispersed phase pressure, *P*_*D*_, was held constant at 1.0 psig. (F) A detailed zoom of the boxed region in (E) with the average rise time *t*_R_, settling time *t*_S_, overshoot *OS*, and accuracy shown.

The ability of the pump to change the pressure of the sealed vessel without outlet flow was evaluated (figure 4(A)). The pressure was both increased and decreased from 1 to 10 psig with step changes ranging from 1.5 to 2.5 psig. These step changes were performed three times during three different operational cycles, in which the entire system was turned off between replicates. Figure 4(B) displays one of these replicates. The set point is increased and decreased from 2.5 psig to 5.0 psig. A detailed zoom of one of the step changes is displayed in figure 4(C) which also displays average *t*_R_, *t*_S_, *OS*, and accuracy values across all step changes and all replicates. The average *OS* for all the step changes was 15.1 ± 6.3%, the average *t*_*R*_ was 0.51 ± 0.21 s, the average *t*_*S*_ was 0.84 ± 0.44 s, and the average accuracy was 0.010 ± 0.006 psi or 0.1% of the full span of pressures.

### 3.2. Pressure regulation during microfluidic drop production

The ability of the pump to change the pressure of a vessel during microfluidic drop production was evaluated (figure 4(D)). One pressure regulator varied *P*_*D*_ from 1.0 to 4.0 psig and another regulator varied *P*_*C*_ from 1.0 to 5.5 psig. This range of pressures was chosen as it yielded drop production in the dripping regime of the microfluidic device [22]. Drops were generated with lengths ranging from 100 *μ*m to 250 *μ*m. To evaluate the performance of the pump, *P*_*D*_ was held constant at 1.0 psig and *P*_*C*_ was increased and decreased by 0.5 psig. The step changes shown in figure 4(E) were performed in triplicate for the three different *P*_*D*_ values used. Each *P*_*D*_ value was set during a different operational cycle. Figure 4(E) shows one of the replicates for step changes of *P*_*C*_. Figure 4(F) shows a detailed section of figure 4(E) where the set point is changed from 2.5 psig to 5.0 psig as well as the average *t*_R_, *t*_S_, *OS*, and accuracy values across all step changes and all replicates. The average *OS* for the changes in *P*_*C*_ was 26.8 ± 4.2%, the average *t*_*R*_ and *t*_*S*_ was 0.74 ± 0.07 s and 1.23 ± 0.16 s respectively, and the average accuracy was 0.016 ± 0.001 psi or 0.3% of the full span of pressures.

There was no significant difference between pressure regulation of the continuous oil phase and dispersed water phase. The fluid being pressurized mainly affects the damping of the pressure regulation, or how much the pressure oscillates [16]. The amount of damping is proportional to the viscosity of the fluids. Since the water and oil phases have similar viscosities, 1 mPa·s and 1.24 mPa·s respectively, both phases have similar dampening effects.

### 3.3. Drop production

**Figure 5.**
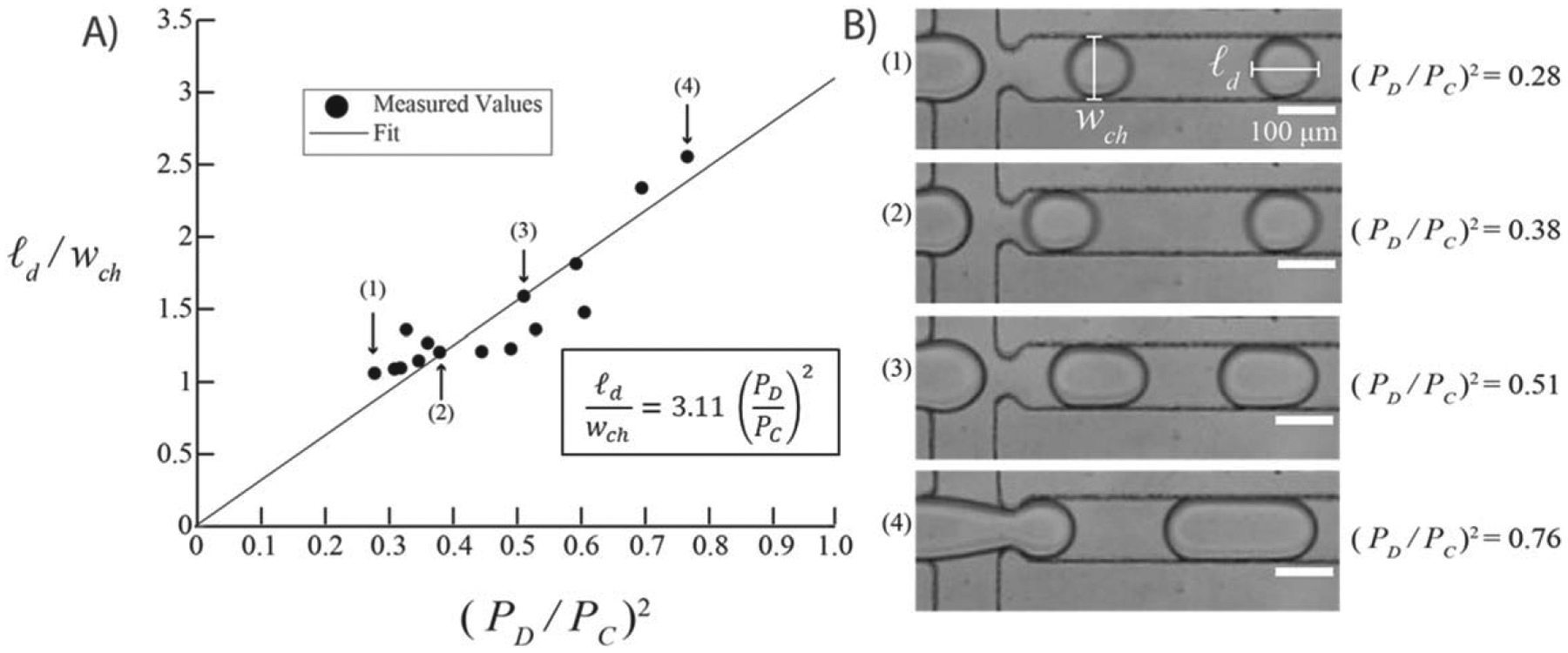
Microfluidic drop production using the pneumatic pressure pump. (A) Drop length *ℓ*_*d*_ normalized by *w*_*ch*_ as a function of applied pressures (*P*_D_/*P*_C_)^2^ using the in-house pressure pump. A fit (solid line) was made using equation 1 with an *α* value of 3.11 (R^2^ = 0.799). Error bars are displayed but are not visible. (B) Selected images of the drop-making process where the dispersed phase extends into the exit channel past the constriction and is pinched off into drops. Scale bars indicate 100 *μ*m.

Finally, we evaluated if the pressure pump can create microfluidic drops of different sizes based on the pressures used to drive microfluidic flow. A plot of *ℓ*_*d*_*/w*_*ch*_ as a function of (*P*_*D*_/*P*_*C*_)^2^, shown in figure 5(A), demonstrates that there is a power-law relationship between the normalized drop length and the pressure ratio that matches the relationship described in past literature [20]. Linear regression was used to determine the value of the scalar constant, *α* in equation (1). *α* was found to have a value of 3.11 with the fit having a *R*^*2*^ value of 0.799, figure 5(A). This *R*^*2*^ value was found to be a reasonable fit for several reasons. Firstly, we tested values of *P*_*D*_/*P*_*C*_ that are larger than those tested by Ward *et al*, and equation (1) appears to have a poorer fit at those values [20]. Additionally, there is no universal scaling law for drop lengths and applied pressures in flow-focusing microfluidic devices [23]. Therefore, we chose an scaling law that captures only the essential parameters during these experiments [24]. These phenomenological models are effective for designing and testing flow-focusing microfluidic devices without relying on more recent machine-learning based models [25]. As expected, drops increase in size as *P*_*D*_ is increased relative to *P*_*C*_. An average of 200 drops were analyzed per condition. Selected still images using brightfield microscopy depict drop production under varying pressure ratios (figure 5(B)).

## 4. Discussion

The pneumatic pressure pump presented here is an advancement towards more open-source versions of these pumps. The pump in this work uses a single Arduino microcontroller to control four purpose-built regulators to provide accurate pressure regulation that can drive flow in microfluidic devices. Frank *et al* is the only comparable pump that did not use an Arduino controller and only Gao *et al* also used a single controller to control four regulators. The only specialty components of the pump are the proportional solenoid valves and the pressure sensors, components which end users can exchange for other versions depending on their specific downstream application. Only Watson and Senyo also used custom dual-valve regulators, all other comparable systems use commercial regulators. We create a GUI in Python, a popular and open-source programming language, while Watson and Senyo and Gao *et al* chose C++. Watson and Senyo’s pump are the most like our pump, with respect to the components used; however, we use our pump to drive flow, while Watson and Senyo actuated microfluidic valves. The components used in comparable pressure pumps is listed in table 1.

Pneumatic pressure pumps have several advantages over syringe pumps and hydrostatic pumps when driving flow in microfluidic devices. Compared to syringe pumps, pneumatic pumps have smaller flow rate fluctuations and faster response times [11], a wider range of usable pressures compared to hydrostatic pumps [5], and more flexibility in the sizes of vessels available. Syringe capacities are limited to smaller volumes, ranging between 1 ml to 50 ml. Additionally, elevating large vessels above small microfluidic devices in the case of hydrostatic pressure pumps may not be practical for all applications. By contrast, silicone septa are available for 15 ml and 50 ml conical tubes and 100 ml–10l glass bottles (figure 2(B)). Even smaller vessels such as 1.5 ml–2 ml microcentrifuge tubes can be sealed with epoxy. All these sealed vessels can be used with pneumatic pressure pumps.

The pressure pump presented here utilizes customizable and easily assembled pressure regulators. The dual proportional valve design is similar to Watson and Senyo’s pump [9], yet is easier to assemble because it does not require a custom manifold and all four regulators are controlled from a single microcontroller. In our system, the solenoid valves use compression fittings upstream and downstream of the valve (figure 2(C)). This allows for the use of hard plastic tubing at these connections followed or preceded by soft tubing to connect the valves to the other pneumatic components. These connections allow users to avoid using custom-machined housings and only requires typical barbed tubing connectors. Additionally, having all the pressure regulators controlled from a single microcontroller eliminates the need to use a bootloader, or an external Arduino programmer, when uploading the control scheme. Each solenoid valve is controlled from a transistor switch with a relatively simple circuit design (figure 1(B)). The circuitry fabrication relies on through-hole soldering which is more accessible for users without a strong background in circuit assembly (figure 2(D)). A simple proportional scheme is used to control the pressure regulators; however, future users could implement another control scheme if desired. The tubing connections and the use of one microcontroller for multiple pressure regulators makes the pressure regulator presented here the most customizable and accessible to-date.

Our pneumatic pressure pump has a higher settling time, *t*_S_, when compared to Watson and Senyo’s regulator [9], and the same *t*_S_ when compared to Gao *et al*’s commercial pressure regulator [10]. The pump presented here has a *t*_S_ of less than two seconds, the *t*_S_ of Watson and Senyo’s regulator was approximately 29 milliseconds [9], and Gao *et al*’s pressure pump had a *t*_S_ of less than two seconds [10]. However, *t*_S_ less than two seconds are adequate for changing the pressures during drop production and other long-term microfluidic processes since frequent pressure changes during microfluidic processes are uncommon once the target drop size is reached. A comparison of the *t*_*S*_ values for previously published pneumatic pressure pumps is presented in table 2.

The pneumatic pressure pump presented here has an improved overshoot and accuracy compared to the pressure regulator from Watson and Senyo [9]. The average overshoot of our pump is 15.1% without flow and 26.8% during drop-making, while Watson and Senyo reported an average of 39.9% with the same range of pressures. The overshoot for our pump could be improved with an inlet pressure less than 14 psig and closer to the maximum pressure used during the drop-making process. The average accuracy of our pump is 0.01 psig without flow and 0.016 psig during drop-making, while Watson and Senyo’s pump reported an accuracy of 0.12 psig with the same range of pressures. The improved accuracy of our experiments could be due to the solenoid valves used or the proportional gain used in the control scheme. Gao *et al* [10], did not report an overshoot percentage. The commercial regulators that Gao *et al* used had an accuracy of 0.03 psig. However, an accuracy of less than 1% of the target pressure is adequate for driving flow in microfluidic devices. A comparison of the *OS* percentage and accuracy values for previously published pneumatic pressure pumps is presented in table 2.

At this time of writing, our pneumatic pressure pump is costlier than the pump published by Watson and Senyo, but is slightly less costly than the pump published by Gao *et al* The costliest components for each of the pumps are the pressure regulators. Our pump uses two proportional solenoid valves that cost $544 for a pair, Watson and Senyo used valves that cost $140 for a pair, and Gao *et al* used commercial regulators that cost $435. Gao *et al* had other components that increased the costs to $750 per regulator, the Watson and Senyo’s pump had a cost of $200 per regulator, and our pump had a cost of $600 per regulator. Comparing the total cost of the pneumatic pressure pumps is only applicable between the pump presented in this work and the pump presented by Gao *et al* since both pumps were both designed to drive flow, while the total cost of the pump presented by Watson and Senyo incorporates the solenoid valves used to actuate the microfluidic valves. Our pump system is slightly cheaper despite using more costly pressure regulators. This cost difference is due to Gao *et al* using different pneumatic connections, an acrylic housing, and different pressure sensors and actuators. Though slightly more expensive, our proportional solenoid valves improved performance with respect to overshoot and accuracy, and the solenoid valves we used are also more easily assembled. The costs of previously published pneumatic pressure pumps is presented in table 2.

The pneumatic pressure pump can create microfluidic drops in a consistent and precise manner. Figure 5(A) shows that a range of drop sizes can be created using the same microfluidic drop maker based upon the ratio of applied pressures (*P*_D_/*P*_C_)^2^. The scaling equation relates the normalized drop length *ℓ*_*d*_/*w*_*ch*_ to the applied pressure ratio (*P*_D_/*P*_C_)^2^. A linear regression was performed that yields a scalar multiplier, *α*, which was 3.11 in this work, comparable to a value of 5.22 in Ward *et al* [20]. The coefficients are of the same order of magnitude, yet vary due to slight differences in device geometries [24]. The drops produced were monodisperse with average variances less than 1%.

## 5. Conclusion

The pneumatic pressure pump presented in this paper is an accurate and accessible pump for driving flow in microfluidic devices. The pump uses an open-source microcontroller and uses a GUI written in the attainable and open source language Python. The pump relies on pressure regulators that utilize a dual proportional solenoid design. A single microcontroller is used to control four pressure regulators with transistor switches. All pneumatic connections use easy-to-assemble barbed, compression, or luer taper tubing fittings contained in a 3D printed enclosure.

The pump presented here can rapidly and accurately change and maintain changes in pressure. The average rise times (*t*_*r*_) and settling times (*t*_*s*_) with or without flow were less than one second and less than two seconds, respectively. The pump had an average overshoot percentage of 27% and was accurate within 0.02 psig, during drop-making. The cost per regulator was approximately $600, which is three times costlier than Watson and Senyo’s regulator, but a quarter of the cost of the commercial regulators used by Gao *et al*. The pump presented in this work is an improvement over open-source pressure regulators; although it had a much slower settling time than Watson and Senyo’s open source pressure regulator, it had an improved overshoot percentage and accuracy. This pump also used the first open-source pressure regulators to drive microfluidic flow, specifically to make microfluidic drops.

The pump presented here was evaluated for its ability to produce drop sizes proportional to the pressures used to drive flow during the drop-making process. The pump was able to produce drops in a flow-focusing microfluidic device whose dimensions are proportional to the pressures used to drive fluid flow, similar to the work published in Ward *et al* [20]. Future directions include the implementation of the pressure pump for microfluidic process that utilize single-phase flow, such as organs-on-a-chip that require long infusion times [26–28], or more complicated drop-based microfluidic processes, such as drop injection, merging, and splitting [29–31]. This pressure pump could also easily operate multiple drop-making devices in parallel with the pressurized reservoirs going to several devices at the same time. We envision applying the pressure pump to improve particle encapsulation into drops, as each pressurized vessel can be easily stirred to prevent the particles from settling in the vessel. Finally, this pump can be used for any number of applications that require precision fluid handling for scientists or engineers at a reasonable cost.

## Figures and Tables

**Table 1.** Comparison of components across published pressure pumps.

Pump	Controller	Regulators	Number of regulators per controller	GUI Language	Application
Presented here	Arduino	Custom	4	Python	Driving flow
Frank *et al* 2016	Commercial Fieldbus	Commercial	1	Python	Driving flow
Watson and Senyo 2016	Arduino	Custom	1	C++	Actuating valves
Gao *et al* 2020	Arduino	Commercial	4	C++	Driving flow

**Table 2.** Comparison of pressure regulation variables across published pressure pumps.

Pump	Tested Pressure Range (psig)	*t*_*R*_(s)	*t*_*S*_(s)	*OS* (%)	Accuracy (psig)	Cost per channel (USD)	Overall Cost (USD)	Number of channels
Presented here	[1,10]	0.74 ± 0.07	1.23 ± 0.16	26.8 ± 4.2	0.016 ± 0.001	602	2,400	4
Frank *et al* 2016	[0,14.5]	N/A	N/A	N/A	0.14 ± 0.04	792	N/A	1
Watson and Senyo 2016	[3,29.5]	0.01 ± 0.01	0.03 ± 0.01	39.9 ± 25.2	0.12 ± 0.06	201	1,730	2
Gao *et al* 2020	[0,14.5]	N/A	< 2	N/A	0.03	500	3,000	4

## Data Availability

The data cannot be made publicly available upon publication because no suitable repository exists for hosting data in this field of study. The data that support the findings of this study are available upon reasonable request from the authors.
